# Active and Secretory IgA-Coated Bacterial Fractions Elucidate Dysbiosis in *Clostridium difficile* Infection

**DOI:** 10.1128/mSphere.00101-16

**Published:** 2016-05-25

**Authors:** Mária Džunková, Andrés Moya, Jorge F. Vázquez-Castellanos, Alejandro Artacho, Xinhua Chen, Ciaran Kelly, Giuseppe D’Auria

**Affiliations:** a Área de Genómica y Salud, Fundación para el Fomento de la Investigación Sanitaria y Biomédica de la Comunidad Valenciana (FISABIO-Salud Pública), Valencia, Spain—Instituto Cavanilles de Biodiversidad y Biología Evolutiva, Universitat de València, Valencia, Spain; b CIBER en Epidemiología y Salud Pública (CIBEResp), Madrid, Spain; c Division of Gastroenterology, Beth Israel Deaconess Medical Center, Harvard Medical School, Boston, Massachusetts, USA; National Institute of Advanced Industrial Science and Technology (AIST), Japan

**Keywords:** 16S rRNA gene sequencing, Bayesian networks, *Clostridium difficile* infection, antibiotics, dysbiosis, fluorescence-activated cell sorting, human gut microbiome, secretory immunoglobulin A

## Abstract

The onset of *Clostridium difficile* infection (CDI) has been associated with treatment with wide-spectrum antibiotics. Antibiotic treatment alters the activity of gut commensals and may result in modified patterns of immune responses to pathogens. To study these mechanisms during CDI, we separated bacteria with high cellular RNA content (the active bacteria) and their inactive counterparts by fluorescence-activated cell sorting (FACS) of the fecal bacterial suspension. The gut dysbiosis due to the antibiotic treatment may result in modification of immune recognition of intestinal bacteria. The immune recognition patterns were assessed by FACS of bacterial fractions either coated or not with intestinal secretory immunoglobulin A (SIgA). We described the taxonomic distributions of these four bacterial fractions (active versus inactive and SIgA coated versus non-SIgA coated) by massive 16S rRNA gene amplicon sequencing and quantified the proportion of *C. difficile* toxin genes in the samples. The overall gut microbiome composition was more robustly influenced by antibiotics than by the *C. difficile* toxins. Bayesian networks revealed that the *C. difficile* cluster was preferentially SIgA coated during CDI. In contrast, in the CDI-negative group *Fusobacterium* was the characteristic genus of the SIgA-opsonized fraction. *Lactobacillales* and *Clostridium* cluster IV were mostly inactive in CDI-positive patients. In conclusion, although the proportion of *C. difficile* in the gut is very low, it is able to initiate infection during the gut dysbiosis caused by environmental stress (antibiotic treatment) as a consequence of decreased activity of the protective bacteria.

**IMPORTANCE** *C. difficile* is a major enteric pathogen with worldwide distribution. Its expansion is associated with broad-spectrum antibiotics which disturb the normal gut microbiome. In this study, the DNA sequencing of highly active bacteria and bacteria opsonized by intestinal secretory immunoglobulin A (SIgA) separated from the whole bacterial community by FACS elucidated how the gut dysbiosis promotes *C. difficile* infection (CDI). Bacterial groups with inhibitory effects on *C. difficile* growth, such as *Lactobacillales*, were mostly inactive in the CDI patients. *C. difficile* was typical for the bacterial fraction opsonized by SIgA in patients with CDI, while *Fusobacterium* was characteristic for the SIgA-opsonized fraction of the controls. The study demonstrates that sequencing of specific bacterial fractions provides additional information about dysbiotic processes in the gut. The detected patterns have been confirmed with the whole patient cohort independently of the taxonomic differences detected in the nonfractionated microbiomes.

## Abstract (precis)

*C. difficile* is a major enteric pathogen with worldwide distribution. Its expansion is associated with broad-spectrum antibiotics which disturb the normal gut microbiome. In this study, the DNA sequencing of highly active bacteria and bacteria opsonized by intestinal secretory immunoglobulin A (SIgA) separated from the whole bacterial community by FACS elucidated how the gut dysbiosis promotes *C. difficile* infection (CDI). Bacterial groups with inhibitory effects on *C. difficile* growth, such as *Lactobacillales*, were mostly inactive in the CDI patients. *C. difficile* was typical for the bacterial fraction opsonized by SIgA in patients with CDI, while *Fusobacterium* was characteristic for the SIgA-opsonized fraction of the controls. The study demonstrates that sequencing of specific bacterial fractions provides additional information about dysbiotic processes in the gut. The detected patterns have been confirmed with the whole patient cohort independently of the taxonomic differences detected in the nonfractionated microbiomes.

## INTRODUCTION

*Clostridium difficile* infection (CDI) is a nosocomial disease associated with broad-spectrum antibiotics, such as clindamycin and fluoroquinolones, but cases without records of previous antibiotic treatment are also regularly reported. CDI is commonly treated with the antibiotic vancomycin or metronidazole (1, 2). At the time of CDI diagnosis in daily clinical practice, some hospitalized patients are already under preventive multiple antibiotic treatments, but some of them do not take any antibiotics at all. This means that at the time of sampling, the gut microbiome of the CDI-positive patients may be disrupted by multiple antibiotics (3, 4). This may be the reason for the results of metagenomics studies comparing CDI-positive and CDI-negative patients being very elusive (5, 6).

The 16S rRNA gene-based analysis and metagenomics also take into account dead or quiescent bacteria present in the collected fecal samples. However, for an exact explanation of dysbiosis mechanisms during CDI, the active bacterial cells must be distinguished from the dead cells. One of the possible mechanisms for opportunistic pathogen invasion is that the antibiotic treatment may alter the activity of certain commensal species, which under normal conditions inhibit the growth of pathogens. An alternative is that some members of the disturbed gut microbiome may become metabolically more active and may start to produce increasing levels of sialic acid, the primary bile acid taurocholate, and carbon sources such as mannitol, fructose, sorbitol, raffinose, and stachyose, which opportunistic pathogens may use for expansion (7, 8). The active bacteria can be distinguished from the inactive bacteria by fluorescent labeling of specific cell targets, e.g., cell wall and intracellular RNA (9–11). Cells growing under optimal conditions have fast cell division and therefore have high intracellular RNA content (12). Such cells are here referred to as “active cells.” In contrast, the cells waiting for the optimal growth condition have low RNA content and are referred to as “inactive cells.”

Moreover, in order to explain the infection processes connected to the dysbiosis, immune system recognition patterns must also be taken into account. The first line of defense in protecting the intestinal epithelium from pathogens is formed by intestinal secretory immunoglobulin A (SIgA), which coats 25 to 75% of all gut bacteria, including the commensal and pathogenic species (13). The SIgA opsonization of commensals and pathogens results in two different outcomes: the first being that the commensal SIgA-coated bacteria are maintained within the gut lumen, and the second being that the SIgA-coated pathogens attempting to cross the epithelial barrier are removed (14, 15). Previous studies revealed that healthy individuals share a core of SIgA-coated bacteria (11, 16). It is not known whether the taxonomic patterns of SIgA-coated bacterial fractions of CDI-positive patients and the CDI-negative control group differ. It was found that antibiotic treatment provides a “blooming” opportunity for specialized pathogens (such as *Neisseria gonorrhoeae* or *Corynebacterium diphtheriae*) which are able to avoid SIgA opsonization by covering their cell surface with molecules (e.g., sialic acids) produced by antibiotic-resistant species (17, 18). It is not known whether such a mechanism exists in CDI.

The microbial composition of the active bacterial fraction and of the fraction coated with SIgA can be determined by 16S rRNA gene sequencing of labeled bacteria selected by fluorescence-activated cell sorting (FACS) (9, 10, 11, 12, 16, 19). We hypothesized that *C. difficile* may in fact be a characteristic species of one of the separated bacterial fractions. The proliferation of *C. difficile* in the intestine may be connected to the increased activity of antibiotic-resistant species or to the eradication of sensitive bacteria. In addition, it is not known whether *C. difficile* is recognized by SIgA as a common pathogen or whether it is able to avoid SIgA opsonization. Our objective was to find statistically relevant associations between the microbial composition of active/inactive and SIgA-coated/non-SIgA-coated fractions and the patients’ medical data.

## RESULTS

The participants in this study were 24 hospitalized patients at the Beth Israel Deaconess Medical Center (BIDMC), Harvard Medical School, Boston, MA, USA. They have been tested by the routine Illumigene assay for CDI, as they either presented symptoms similar to CDI or fell within the CDI risk category. CDI was diagnosed in 12 of these patients, while 12 were CDI negative, as shown in Table 1.

**TABLE 1:** Patient medical data^a^

Patient no.	Patient designation	Frequency of gene as determined by qPCR			Antibiotic(s) (concn)		
		16S rRNA genes from *C. difficile*	Toxin A	Toxin B	Used for CDI treatment	Associated with onset of CDI	With no reported association with CDI
1	CDIneg01				Vancomycin (2 g), metronidazole (12 g)		Tigecycline (0.05 g)
2	CDIneg02				Vancomycin (7 g)	Cefepime (54 g)	
3	CDIneg03	0.0002			Vancomycin (6 g)	Cefepime (12 g)	Cefazolin (6 g)
4	CDIneg04	0.0008			Vancomycin (12 g), metronidazole (9 g)	Cefepime (36 g)	Ampicillin-sulbactam (48 g), amoxicillin-clavulanic acid (3.5 g)
5	CDIneg05						
6	CDIneg06	0.0002					
7	CDIneg07	0.0001			Rifaximin (0.25 g)	Ciprofloxacin (0.25 g)	
8	CDIneg08				Vancomycin (1.25 g)	Cefepime (2 g)	
9	CDIneg09				Metronidazole (5 g)	Ciprofloxacin (5 g)	
10	CDIneg10						
11	CDIneg11						
12	CDIneg12						Piperacillin-tazobactam (9 g)
13	CDIpos01	0.0972	0.0012	0.0012			
14	CDIpos02	0.1091	0.0003	0.0010			
15	CDIpos03	0.8580	0.0055	0.0054	Metronidazole (12 g), vancomycin (16 g)	Ciprofloxacin (7.2 g)	
16	CDIpos04	0.0013	0.0010	0.0012			
17	CDIpos05	0.0044	0.0002	0.0010			
18	CDIpos06	0.8881	0.0071	0.0101			
19	CDIpos07	0.0262	0.0002	0.0001	Vancomycin (2 g)	Ceftriaxone (2 g), cefepime (6 g)	Piperacillin-tazobactam (27 g), amoxicillin-clavulanic acid (5 g)
20	CDIpos08	0.0406	0.0004	0.0010			
21	CDIpos09	0.0021	0.0002	0.0001			
22	CDIpos10	0.0029	0.0002	0.0010		Ciprofloxacin (1 g)	Nitrofurantoin (1.6 g), cephalexin (3 g), piperacillin-tazobactam (27 g)
23	CDIpos11	0.0001	0.0001	0.0001		Levofloxacin (0.5 g)	
24	CDIpos12	0.0284	0.0002	0.0010		Clindamycin (7.2 g)	

a The samples are named CDIneg or CDIpos according to the diagnosis of CDI by the Illumigene assay. The frequency of *C. difficile* in the fecal bacterial suspension was quantified by qPCR, as well as the load of cells containing toxin A and toxin B genes. The antibiotics are divided into three groups according to their relationship to CDI. The total antibiotic dose is shown.

The quantitative PCR (qPCR) assay detected specific *C. difficile* 16S rRNA gene sequences in 16 samples of our cohort; it formed 0.0001% to 0.881% of total gut microbiota (Table 1). Four of these patients were considered clinically CDI negative by an Illumigene assay, and the absence of toxin genes was also confirmed by a qPCR targeting *C. difficile* toxins A and B, indicating that these patients were colonized by nontoxigenic *C. difficile* strains. In six CDI-positive patients, the burdens of toxin A genes quantified by qPCR were 1.4 to 5 times lower than burdens of toxin B genes (Table 1).

**FIG 1:**
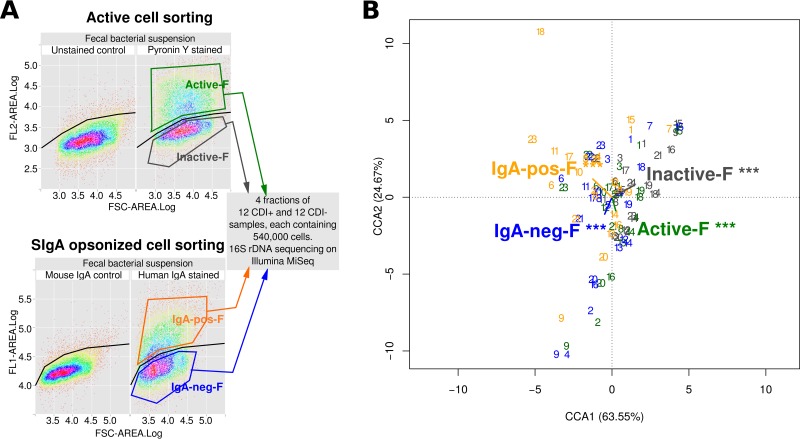
Cell sorting scheme and its impact on bacterial diversity of the samples. (A) FACS biplots showing setup of sorting gates in comparison with negative controls. Each sample was used in two separated sorting rounds: (i) active-bacterium sorting and (ii) IgA-coated-bacterium sorting. For each fraction, 540,000 cells were separated and 16S rRNA genes were amplified and sequenced. FSC, forward scatter. (B) Canonical correspondence analysis of ordination of fractionated fecal samples by fitting their overall bacterial composition into variables of active-F, inactive-F, IgA-pos-F, and IgA-neg-F. The fractionated samples are numbered 1 to 24 and are colored in accordance with the fraction colors. Analysis showed that the fractions have a significant influence on the bacterial composition, meaning that the differences among samples belonging to different fractions may be detected in the subsequent analysis.

The aliquots of fecal bacterial suspensions of the 24 samples were processed in two subsequent rounds of cell sorting: (i) SIgA-coated versus non-SIgA-coated bacteria and (ii) active versus inactive bacteria (Fig. 1A). The resulting fractions have been designated IgA-pos-F, IgA-neg-F, active-F, and inactive-F, respectively. The counting of the fluorescently labeled cells showed that the average proportion of cells belonging to the IgA-pos-F fraction was lower (39.67% ± 7.73%) than the average proportion of the active-F fraction (63.02% ± 5.56%) (see Fig. S1 in the supplemental material). When CDI-positive and CDI-negative groups were compared and, similarly, when antibiotic-positive and antibiotic-negative groups were compared, no significant differences in proportions of cells belonging to the IgA-pos-F fraction and the active-F fraction were detected (*P* > 0.01) (see Fig. S2).

10.1128/mSphere.00101-16.1 Figure S1  Proportion of events in FACS gates. (A) Proportion of events (percent) in the FACS gate for cells not stained by IgA, in the gate for mouse-IgA isotype control, and in the gate for human IgA staining. The mouse-IgA control removed nonspecifically labeled bacteria (31.05% ± 2.43% of all bacteria) that would be counted within the IgA-positive fraction if this control were not used. (B) Proportion of events in the gates for active-F and inactive-F fractions. Download Figure S1, PDF file, 0.5 MB. Copyright © 2016 Džunková et al. 2016 Džunková et al. This content is distributed under the terms of the Creative Commons Attribution 4.0 International license.

10.1128/mSphere.00101-16.2 Figure S2  Proportion of cells in separated fractions for samples divided into CDI-positive/CDI-negative and antibiotic-positive/-negative groups. (A) Proportion of active cells. (B) Proportion of bacterial cells belonging to the fraction opsonized by secretory IgA. Download Figure S2, PDF file, 0.1 MB. Copyright © 2016 Džunková et al. 2016 Džunková et al. This content is distributed under the terms of the Creative Commons Attribution 4.0 International license.

The 16S rRNA gene amplicons of the separated fractions have been sequenced. The overall bacterial compositions of the 4 fractions of the 24 samples were analyzed together in the canonical correspondence analysis (Fig. 1B). The analysis detected differences between the microbial compositions of the active-F, inactive-F, IgA-pos-F, and IgA-neg-F fractions of the 24 patients (*P* < 0.001).

**FIG 2:**
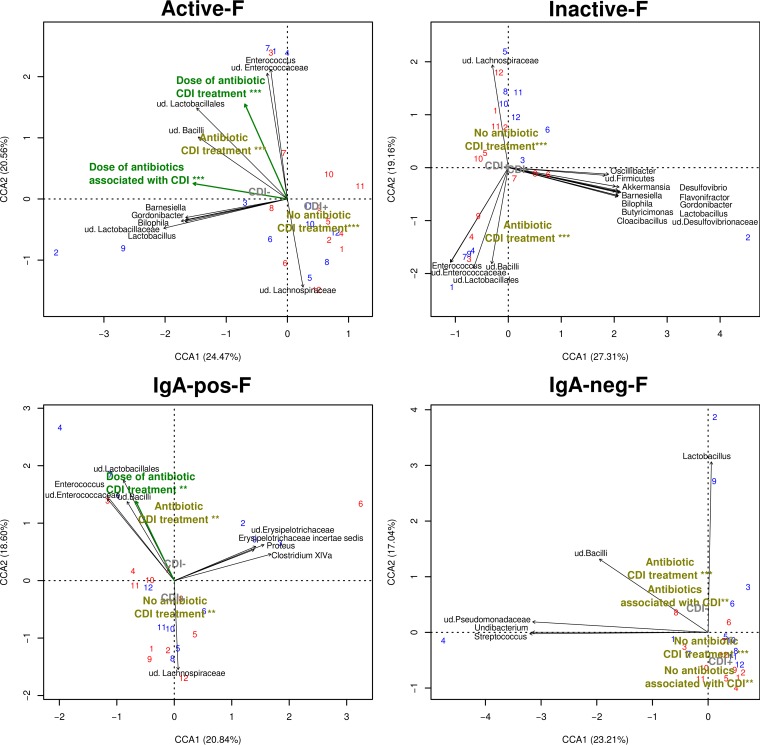
Impact of medical data on bacterial diversity of fractionated samples. A canonical correspondence analysis was undertaken, whereby the bacterial composition was tested to assess the fit with medical data. The analysis was performed separately for each fraction (active-F, inactive-F, IgA-pos-F, and IgA-neg-F). The numerical factors taken into account were the total dose of antibiotic treatment and the loads of toxin A and toxin B genes quantified by qPCR. The categorical medical data taken into account were the diagnosis of CDI and the type of antibiotic treatment ([i] antibiotic against CDI, [ii] antibiotics promoting CDI, and [iii] antibiotics with neutral effect on CDI onset—not associated with CDI). The bacterial species (black) and numerical variables (green boldface) with a significant influence on the ordination of samples are shown (*P* < 0.01). The categorical medical data (olive green boldface) are shown with asterisks corresponding to the *P* value (**, *P* < 0.01; ***, *P* < 0.001). The diagnosis of CDI as a categorical factor did not have a significant influence on the ordination of samples (*P* > 0.05); its ordination effect is shown in light gray for illustration purposes only. Samples are marked by numbers in red or blue corresponding to CDI-positive or CDI-negative patients, respectively. ud., undetermined taxon.

In the subsequent canonical correspondence analysis (Fig. 2), the overall bacterial composition of each of the four fractions was tested to assess the fit with patients’ medical data (CDI diagnosis and type of antibiotic treatment). Three antibiotic categories have been taken into account: (i) antibiotics for treatment of CDI, (ii) antibiotics associated with the onset of CDI, and (iii) antibiotics with no reported association with CDI. Antibiotics were found to significantly shape the overall bacterial composition of the four separated fractions (*P* < 0.05). In contrast, the diagnosis of CDI positive or negative and the quantified amounts of toxin A and toxin B did not have a significant influence on the overall bacterial composition of the bacterial fractions. These findings indicate that the antibiotics have a greater influence on the composition of the microbiota than the *C. difficile* toxins. The microbiome of patients undergoing antibiotic treatment differed from those patients that did not take any antibiotics. The microbiome of the antibiotic-free group was characterized by the presence of the *Lachnospiraceae* family. *Enterococcus* was typical in patients undergoing antibiotic CDI treatment (metronidazole and vancomycin) in the four fractions. These findings demonstrate that a certain portion of cells belonging to a bacterial genus is active, while another portion is inactive (and the same is true for SIgA coating).

In the subsequent analysis, we compared the proportions of cells belonging to a single genus found in either active or inactive bacterial fractions. In order to simulate the decrease of bacterial activity in the presence of antibiotics *in vitro*, we performed batch culture experiments (see Fig. S3 in the supplemental material). Fecal suspensions were cultivated in the gut culture medium (20) with or without metronidazole. Samples with an 0.5-ml volume were taken every hour from the batch culture, fixed by formaldehyde, and fluorescently stained for cellular RNA. The monitoring of growth by flow cytometry showed that the bacterial culture with metronidazole grew more slowly than the culture without antibiotics. Slowed cell division in culture with metronidazole permitted us to spot two separate populations of cells with low or high intracellular RNA content (inactive-F and active-F). The 16S amplicon sequencing showed that the active-F fraction of the culture without metronidazole was characterized by an overgrowth of *Escherichia/Shigella* (76.2%)*.* In contrast, the culture with metronidazole contained a major proportion of *Enterococcus* in the active-F fraction after 10 h (76.2%). However, a small portion of *Enterococcus* was also found in the inactive-F fraction (12.5%). This is in accordance with the results observed for the patients of our cohort undergoing metronidazole treatment.

10.1128/mSphere.00101-16.3 Figure S3  Results of 16S rRNA gene sequencing of microbial culture sample cultivated with and without antibiotics. One milliliter of the fecal bacterial suspension was inoculated in 10 ml of the anaerobic medium reported to recover a wide diversity of human gut microbiota (20). The medium contained either no antibiotics or metronidazole (40 mg/liter). Culture samples with an 0.5-ml volume were taken every hour from each flask, stained for bacterial activity, and visualized on a flow cytometer, as shown in this figure. The active bacterial fractions of the time points 0 h and 10 h were separated by FACS. The 16S rRNA gene amplicons of the active fractions have been sequenced. The results of taxonomic assignation are shown below the flow cytometry plots. Download Figure S3, PDF file, 0.3 MB. Copyright © 2016 Džunková et al. 2016 Džunková et al. This content is distributed under the terms of the Creative Commons Attribution 4.0 International license.

**FIG 3:**
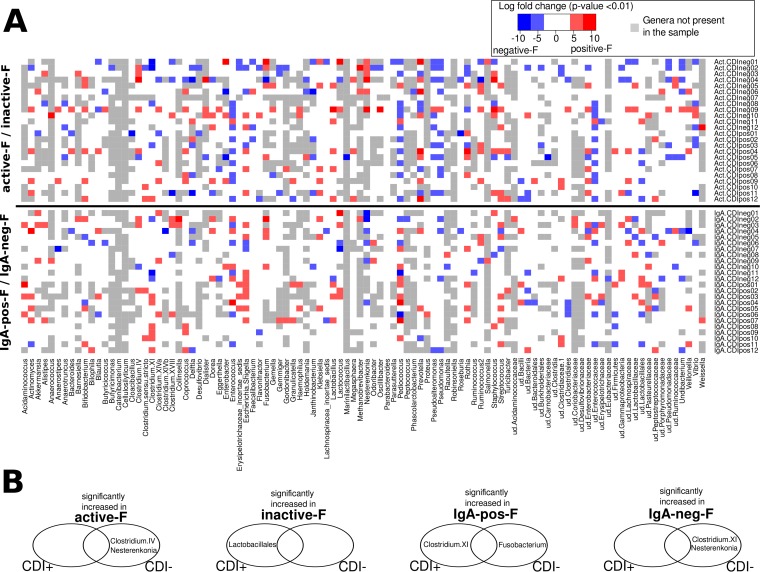
Fold change differences for each bacterial genus in the two fraction pairs. (A) Heat map showing comparison of proportion of each genus in the active-F fraction with its proportion in the inactive-F fraction, as well as its proportion in the IgA-pos-F fraction compared with its proportion in the IgA-neg-F fraction. Genera significantly increased (*P* < 0.01) in active-F or IgA-pos-F fractions are marked in red, while the genera significantly increased in inactive-F or IgA-neg-F fractions are marked in blue. The intensity of red or blue depends on the fold increase. The white fields show that there was no significant increase in any of the compared fractions. The gray fields show that a genus was not detected in the sample at all. The samples and the bacterial genera are ordered alphabetically. ud., undetermined taxon. (B) The results shown in panel A have been tested for their cooccurrence with medical data in a Bayesian network. Summary of the strongest associations obtained by network modeling. The Venn diagrams show which bacterial genera were found to have significantly increased proportions in one of the four separated fractions compared with their fraction counterparts. CDI-positive and CDI-negative groups had different patterns of bacterial activity and SIgA coating.

The above-mentioned analysis showed that the majority of the detected bacterial genera were found in both active-F and inactive-F fractions and also in IgA-pos-F and IgA-neg-F fractions in the patient cohort, but in different proportions. When the proportion of bacterial genera in the active-F fraction was compared with that in the inactive-F fraction and, similarly, when the proportion of genera in the IgA-pos-F fraction was compared with that in the IgA-neg-F fraction, significant fold change differences (*P* value <0.01) were detected (Fig. 3A). The proportion of inactive cells belonging to the genera *Bifidobacterium*, *Lactobacillus*, and *Nesterenkonia* was usually lower than the proportion of active cells belonging to these genera. In patients without antibiotic CDI treatment (metronidazole or vancomycin), a significant number of *Enterococcus* cells were inactive. In contrast, in the patients taking metronidazole or vancomycin a greater part of *Enterococcus* cells were actually active. This is consistent with the results of the batch culture experiments.

The aim of the subsequent step was to determine whether the medical data influence the proportions of cells of a selected bacterial genus detected either in the active-F fraction or in the inactive-F fraction (and also either in the IgA-pos-F fraction or in the IgA-neg-F fraction). The statistically significant associations of the medical data (the three types of antibiotic treatment and the CDI diagnosis) with the data visualized in Fig. 3A have been established by a Bayesian network model. From the obtained network, we extracted the information on how medical data predict the patterns of bacterial activity and SIgA coating (Markov blanket dissection; see Materials and Methods). The results of this analysis are summarized in Fig. 3B; they indicated that the patterns of bacterial activity and SIgA opsonization differed among CDI-negative and CDI-positive patients. In CDI-positive patients, a greater portion of the cells belonging to the order *Lactobacillales* were inactive. In contrast, this was not observed in the CDI-negative cohort, who had significantly increased *Nesterenkonia* and *Clostridium* cluster IV cells in the active-F fraction. *Nesterenkonia* was also found significantly increased in the IgA-neg-F fraction in the CDI-negative cohort. *Clostridium* cluster XI (the cluster to which *C. difficile* belongs [21]) was also the typical bacterial group of the IgA-neg-F fraction in the CDI-negative cohort. This is in contrast to the SIgA opsonization patterns of CDI-positive patients, in which a significant majority of *Clostridium* cluster XI cells were found in the IgA-pos-F fraction. *Fusobacterium* was a typically increased genus in the IgA-pos-F fraction of the CDI-negative group.

The patients who were taking antibiotics associated with CDI onset (clindamycin or fluoroquinolones) were diagnosed as either CDI positive or CDI negative. Those patients who were CDI negative and were on antibiotic treatment associated with CDI had significantly increased proportions of active cells of the *Clostridium* cluster IV. The patients who did not take any antibiotics associated with the onset of CDI had in common increased activity of *Rothia* and decreased activity of *Janthinobacterium* cells.

## DISCUSSION

Although *C. difficile* colonization in feces is detectable by qPCR or culture methods, *C. difficile* usually forms less than 1% of all bacterial gut genera. In addition, the healthy gut may also be colonized by nontoxigenic *C. difficile* (22–24), which was also observed in the present study. For this reason, *C. difficile* does not usually appear as a species differentiating CDI-positive and CDI-negative patients in metagenomic analyses (3, 4, 5, 25). The literature reviews showed that a general definition of the microbiome typical for CDI by common metagenomic approaches is elusive (5). Recent studies indicated that CDI onset is probably due to microbiome dysbiosis, which results in the loss of the protective species belonging to the *Clostridiales* order (26, 27). The fractionation of the total bacterial community by FACS performed in this study also helped to elucidate processes occurring during dysbiosis.

The results of fluorescent cell counting indicated that the proportion of active bacteria and bacteria coated with SIgA is very variable, in accordance with previous studies (9, 11, 13, 16, 19). Significantly decreased proportions of active cells in those patients undergoing antibiotic treatment were not observed, which might be a consequence of the replacement of dying bacteria with antibiotic-resistant bacteria. Multi-omics studies (metagenomics, metatranscriptomics, and metabolomics) showed that antibiotic treatment changes the taxonomic distribution of the gut microbiome but that the newly established community continues to maintain the previous metabolic functions of the gut (28), which supports our observation that the activity of the entire bacterial community continues on the same level after antibiotic treatment.

The gut microbiota modulation by *C. difficile* toxins has already been reported (3, 29), but this is the first study in which the influences of *C. difficile* toxins and antibiotics on microbiota are compared. The analysis of the overall bacterial composition of the individual separated fractions showed that the robust influence of antibiotics on the gut microbiota is even stronger than the influence of *C. difficile* toxins on gut microbiota.

Each bacterial genus consists of both active and inactive cells; however, their proportions depend on the antibiotic treatment. For example, the cells of the vancomycin- and metronidazole-resistant *Enterococcus* were mostly found in the active-F fraction of the patients treated with these antibiotics; however, a small portion of *Enterococcus* cells was found also in the inactive-F fraction. In contrast, in the patients with no vancomycin and metronidazole treatment, *Enterococcus* was mostly inactive. This is in accordance with the previous studies, which also showed that a great part of the most dominant genera of the healthy human gut is actually inactive (9, 10).

In the present study, a majority of beneficial *Lactobacillales* and *Clostridium* cluster IV cells were found dying in CDI-positive patients. This is in accordance with previous studies which demonstrated inhibitory effects of *Lactobacillus* on *C. difficile* growth (30). *Clostridium* cluster IV also contains species maintaining gut homeostasis (31). These findings suggest that the growth of *C. difficile* may be suppressed under normal conditions by these beneficial bacteria, while the decrease of their activity due to antibiotics induces a “blooming” of *C. difficile*. This process has already been described for other pathogens (32).

It has been previously reported that only a part of a single bacterial genus is coated with SIgA, while the other part remains uncoated, which may be a reflection of a strain-specific SIgA coating (19). The same comparison of the proportions of cells of a given genus in the IgA-pos-F and IgA-neg-F fractions has been made in this study, which helped to identify patterns of SIgA coating in CDI-positive patients. We detected that the intestinal immune system in CDI-positive patients recognizes *C. difficile*. These findings suggest that this species is not able to avoid SIgA opsonization, as a few other pathogens do (17, 18). In the CDI-negative cohort, it is not *C. difficile* but other bacteria, such as *Fusobacterium*, that have been typically found to be increased in SIgA-coated fractions. Despite the fact that in some studies the presence of *Fusobacterium* was associated with various diseases, *Fusobacterium* also belongs to the most prevalent genera of the gut of healthy individuals (33).

In conclusion, the excessive coating of *C. difficile* (*Clostridium* cluster XI) with intestinal SIgA and the decreased activity of the beneficial bacteria from *Clostridium* clade IV and the *Lactobacillales* order have been identified as markers of CDI. These patterns have been detected across the entire CDI-positive group by the comparison of the proportions of active/inactive cells and SIgA-opsonized/non-SIgA-opsonized cells for each bacterial genus by 16S rRNA gene sequencing of the fractionated gut microbiome, independent of the type of antibiotic treatment being given. On the other hand, the overall gut bacterial composition in CDI patients is mostly shaped by antibiotics, and the influence of antibiotics is even greater than the effect of *C. difficile* toxins.

## MATERIALS AND METHODS

### Patients’ medical data.

The study included 24 patients hospitalized at the Beth Israel Deaconess Medical Center (BIDMC) Division of Gastroenterology (Boston, MA, USA) during the period between 29 May and 19 June 2014, with CDI symptoms or within the CDI risk category. The only exclusion criterion was a fecal sample volume below 5 ml. All 24 patients have been tested for CDI by a routine Illumigene assay (Meridian Biosciences, Cincinnati, OH; catalog no. 280050), with only 12 of them having been diagnosed as CDI positive. The other 12 patients were considered controls in this study. The study was approved by the BIDMC’s institutional review board, and informed consent was obtained from all patients. The CDI diagnosis, sample collection, and initial sample processing have been carried out according to BIDMC guidelines. The samples were further processed according to FISABIO-Public Health (Valencia, Spain) guidelines.

The amounts of *C. difficile* A and B toxins, as well as the 16S rRNA gene of *C. difficile*, were quantified by qPCR (Qiagen, Venlo, Netherlands; catalog numbers BPVF00463AF, BPVF00464AF, and BBID-1506Zy-4).

### Fractions of the total bacterial population.

Fecal samples were processed as described previously (9, 11, 16). The fecal bacterial suspensions were divided into 4 tubes; all of these were labeled with a DNA stain (SYTO 62; Life Technologies, Carlsbad, CA; catalog no. S11344) to distinguish the bacteria from the cytometer electrical noise. The second staining was undertaken using one of the following: IgA-human or IgA-mouse (as an isotype control; Life Technologies; catalog numbers A24459 and M31001) or pyronin Y (staining RNA for active cell sorting; Sigma-Aldrich, Dorset, United Kingdom; catalog no. P9172-1G), while the fourth tube, representing the negative control, remained stained with SYTO 62 only. The aliquots of the fecal bacterial suspensions of the 24 samples were processed in two subsequent rounds of cell sorting (active-cell sorting and IgA-coated-cell sorting), as described for Fig. 1A. Each tube finally contained 540,000 FACS-separated cells (Fig. 1A). The proportion of cells in bacterial fractions was calculated using the “flowViz” package from the R statistics environment.

### Sequencing of 16S rRNA gene of separated fractions.

DNA from all the 96 samples (active-F, inactive-F, IgA-pos-F, and IgA-neg-F fractions for each of the 24 patients) was extracted at one time by phenol-chloroform extraction under sterile conditions. The V3 and V4 regions of 16S rRNA genes (34) were amplified and prepared for sequencing in one MiSeq Illumina run. In order to confirm the results, replicates of the 24 samples were sequenced.

A sequence quality assessment was carried out using the PRINSEQ program (35). Sequences of <200 nucleotides (nt) in length were not considered; 5′ trimming was performed by cutting out nucleotides with a mean quality of <30 in 20-bp windows. Eventual chimeric 16S amplicons were removed by the USEARCH program (36). The final data set resulted in an average of 163,519 sequences per sample.

### Analysis of overall bacterial composition.

Obtained sequences were taxonomically classified by the RDP classifier (bootstrap cutoff, 0.8) program from the Ribosomal Database Project up to the genus taxonomic level (37). Genera represented in fewer than 10 reads on average among all samples were not considered.

Canonical correspondence analysis was used for ordination of the 24 samples (for each fraction separately) in which the bacterial composition was tested to assess the fit with medical data using the “envfit” function from the “vegan” R package. The categorical medical data considered were CDI-positive or -negative diagnosis by the Illumigene assay and the type of antibiotic treatment (categorized into [i] antibiotic against CDI, [ii] antibiotics promoting CDI, and [iii] antibiotics with a neutral effect on CDI onset). The amounts of *C. difficile* toxin A and toxin B and the total doses of each of the three antibiotic types (shown in Table 1) were considered the numerical data.

### Comparison of frequencies of bacterial genera in separated fractions.

Fold change frequency tests between (i) active-F and inactive-F fractions and between (ii) IgA-pos-F and IgA-neg-F fractions were performed with the R package “edgeR.” Bayesian networks were used to find statistically significant associations of the medical data with the statistically significant fold change increase of bacterial genera in one of the compared fraction pairs (*P* < 0.01, Benjamini-Hochberg correction) using the R package “bnlearn.” The nodes of the network represented the preferential occurrence of bacterial genera in the four fractions, the CDI diagnosis, and the three types of antibiotic treatment (excluding any antibiotics started on the day of sample collection). Only bacterial genera that significantly increased in >5 patients in one of the compared fraction pairs were included in the network. The connecting arcs of the network represented a mutual association rather than causality. From a Bayesian network, a subset called Markov blanket can be extracted; it determines which bacteria predict the behavior of a selected node representing medical data (38). In our case, the nodes corresponding to the three types of antibiotics and CDI were dissected.

### Nucleotide sequence accession number.

Sequences have been deposited in the European Nucleotide Archive database under accession no. PRJEB8416.
