# Supplementary figures and images for: Active and Secretory IgA-Coated Bacterial Fractions Elucidate Dysbiosis in Clostridium difficile Infection
Source: mSphere. 2016 May 25;1(3):e00101-16. doi: 10.1128/mSphere.00101-16 (PMC4888886; doi:10.1128/mSphere.00101-16)

(A)

## SlgA-opsorized cell sorting

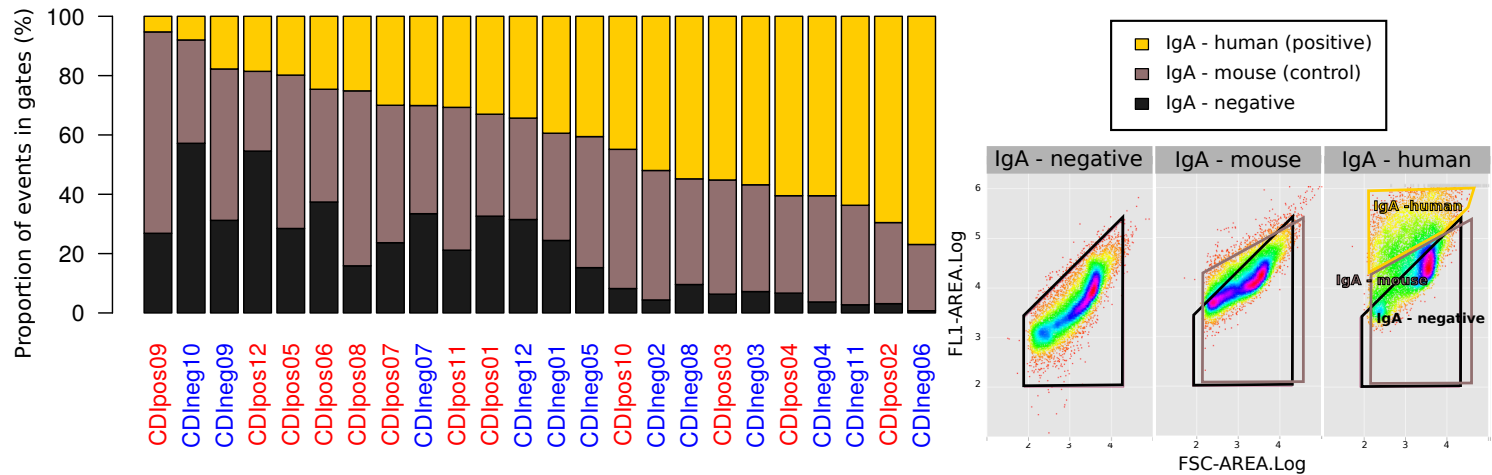

(B)

## Active cell sorting

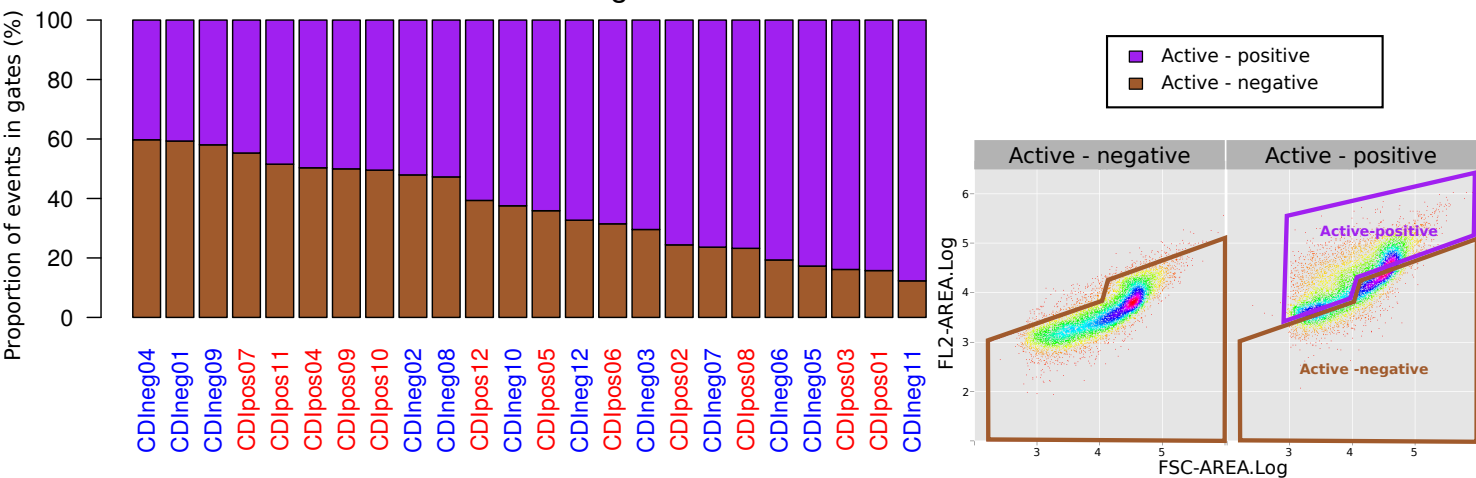

Supplement: Figure S1 [file sph003162091sf1.pdf]

**A****CDI+/-****Antibiotics+/-****Proportion of active-F (%)**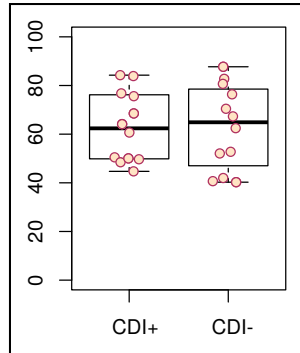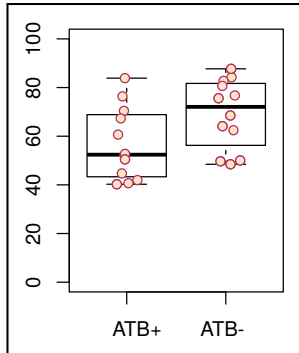**B****CDI+/-****Antibiotics+/-****Proportion of IgA-pos-F (%)**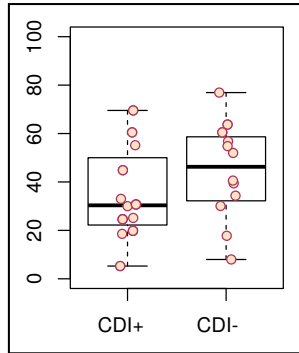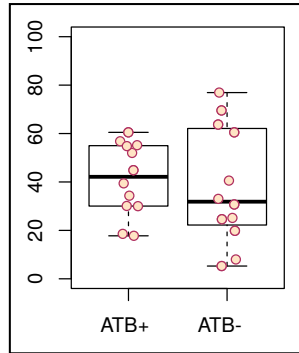

Supplement: Figure S2 [file sph003162091sf2.pdf]

# Culture with metronidazole (40 mg/l)

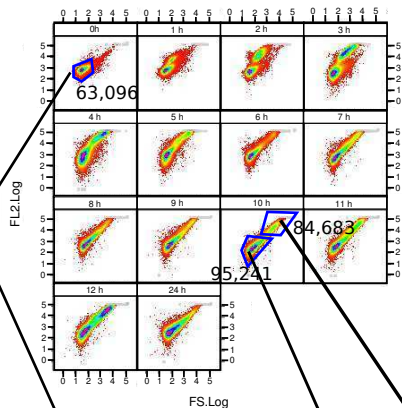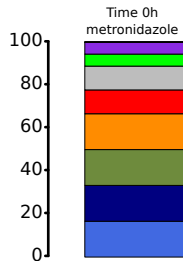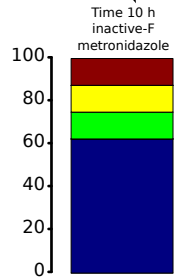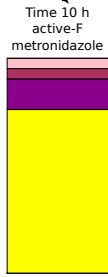

# Culture without antibiotics

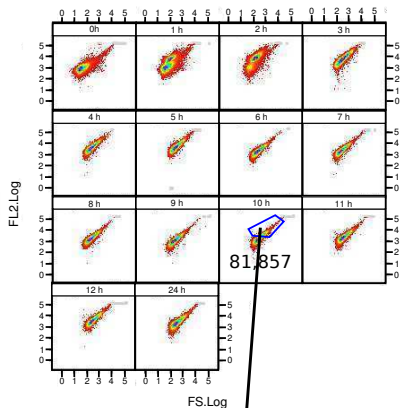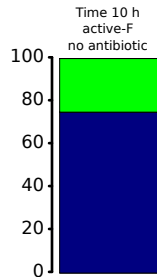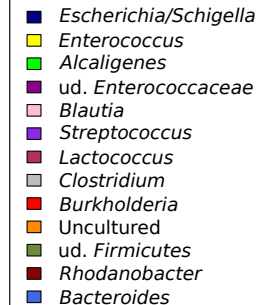

Supplement: Figure S3 [file sph003162091sf3.pdf]
